# Thermobiological effects of temperature‐induced color variations in *Aglais urticae* (Lepidoptera, Nymphalidae)

**DOI:** 10.1002/ece3.8992

**Published:** 2022-06-11

**Authors:** Gregor Markl, Shannon Ottmann, Tobias Haasis, Daniela Budach, Stefanie Krais, Heinz‐R. Köhler

**Affiliations:** ^1^ Department of Geosciences University of Tübingen Tübingen Germany; ^2^ Animal Physiological Ecology Group Institute of Evolution and Ecology University of Tübingen Tübingen Germany

**Keywords:** climate, coloration, Lepidoptera, melanism, physiological effect, selection, temperature, thermobiology

## Abstract

Coloration of animals is important for camouflage, for social behavior, or for physiological fitness. This study investigates the color variation in adults of *Aglais urticae* obtained on subjecting some pre‐imaginal stages to different temperature conditions and their thermobiological consequences. To investigate the evolutionary–ecological interactions of temperature and pigmentation in butterflies, caterpillars, and pupae of the small tortoiseshell, *Aglais urticae* (Lepidoptera, Nymphalidae), larvae from Central Europe and Scandinavia were reared at temperatures between 7 and 34°C in the laboratory or in the field. After emergence, the intensity of pigmentation of the imagines and their increase in body temperature under defined full‐spectrum light irradiation were quantified by image analysis and thermal imaging. At constant conditions, ambient rearing temperature and pigmentation intensity of imagines were negatively and linearly correlated in Central European butterflies, regardless of whether the pupal stage alone or, additionally, the last period of the larval stage was exposed to these conditions: low temperatures induced darker coloration and high temperatures led to lighter individuals. A thermal pulse of a few days alone at the beginning of pupal dormancy led to a similar, albeit weakened, effect. Caterpillars of the Scandinavian subspecies *A*. *urticae polaris*, whose pupal dormancy took place under Central European field conditions, developed into strongly pigmented imagines. The thermobiological relevance of more intense pigmentation was shown by significantly higher absorption of light, and thus stronger increased body temperature after 5 min of defined illumination, but this difference ceased after 15 min. Our results show that phenotypic plasticity in wing coloration is adaptive since temperature‐induced developmental changes provide thermobiological benefit in adult butterflies. We propose that, in subpolar latitudes, darker coloration likely has a selection advantage favoring individuals with reaction norms gradually shifted to stronger pigmented phenotypes, possibly leading to the establishment of a pigmentation cline.

## Graphical abstract

Coloration of animals is important for camouflage, for social behavior or for physiological fitness. *Aglais urticae* butterflies reared at temperatures between 7 and 34°C show at constant conditions a negative correlation between ambient temperature and pigmentation intensity. Darker imagines show a stronger increased body temperature after 5 min of defined illumination which provides an ecological advantage.

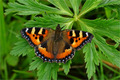

## 1 INTRODUCTION

The thermobiological implications of varying degrees of pigmentation of organisms have often been assumed from theoretical considerations, but have rarely been quantified in a non‐contact manner. This lack is in contrast to the fact that such measurements are indispensable if decisions are to be made about the (thermo)biological relevance of different intensities of coloration and thus any selection pressures that may occur. In this context, the relationship between coloration and temperature can be mutually dependent. On the one hand, absorption of electromagnetic radiation can be intensified by increased pigmentation, but on the other hand, different temperatures prevailing in the individual developmental stages of an organism can also cause different coloration intensities in adult animals.

Dorfmeister (1863–65) was probably the first to realize the importance of ambient developmental temperature on the coloration of adult butterflies, and he also performed the first experiments on this topic. However, it was Standfuss (1900, 1901) who demonstrated temperature‐induced variation in body coloration in tens of thousands of individuals (=pupae) belonging to 56 species of all large butterfly and moth families. He claimed that the most important step during pigmentation pattern development occurs around the transition from larva to pupa, i.e., in a temporal window of development during the last 1 or 2 days of the larval stage and during the first few days of the pupal stage.

Subsequently, butterflies of the family Nymphalidae have been investigated in great detail to understand the formation and location of “colour centers” on their wings during ontogeny, the determination of the final coloration during metamorphosis, and the evolutionary selection of colors in the course of phylogeny (e.g. Hiyama et al., 2012; Kuhn, 1926; Otaki, 2008). Davis et al. (2012) reported that wing color is related to flight performance in Monarch butterflies (*Danaus plexippus*). To date, numerous publications have reported on the responses of wing size and wing color to temperature changes in butterflies (e.g. Bergmann, 1952; Burgeff & Fetz, 1968; Ellers & Boggs, 2004; Forster & Wohlfahrt, 1977; Guppy, 1986; Hegna et al., 2013; Hofmann & Tremewan, 2017; Kennelly et al., 2017; Kertész et al., 2017; Kettlewell, 1973; Krodel, 1904; Kuhn, 1926; Merrifield, 1894; Nijhout, 1984; Rawlins, 1980; Roland, 1982; True, 2003; Xing et al., 2016) and on darker pigmentation in larvae reared in particularly cool temperatures (Goulson, 1994; Hazel, 2002; Hofmann & Tremewan, 2017; Solensky & Larkin, 2003). For example, Hegna et al. (2013) showed that hindwing color of the wood tiger moth *Arctia plantaginis* varies with climatic zone between the Baltic States and Finland. Burgeff and Fetz (1968) reported that in some areas along the Ligurian coast interpreted as glacial refugia, some *Zygaena* species show conspicuous melanistic forms. According to Kennelly et al. (2017), the wing size of *Vanessa cardui* is negatively correlated with temperature, for unexplained reasons. Guppy (1986) showed that more melanistic *Parnassius phoebus* individuals spent more time flying than less melanistic ones. Similar results were also obtained by Roland (1982) who could additionally show that under warmer climate conditions, lighter colored individuals were more active and, hence, had an ecological advantage. In a series of experiments on two mountain Colias species, Ellers and Boggs (2004) showed that male flight activity was strongly correlated with melanism (positively at high altitudes and negatively at low ones), which was, however, not true for females. More melanistic females, on the other side, showed higher egg maturation rates at high altitudes and, hence, gained a significant ecological advantage from stronger melanism.

Some authors investigated the natural variations in butterfly subspecies as a response to different climates (e.g., Harmer & Russwurm, 2000; Hofmann & Tremewan, 2017; Lorkovic, 1943; Shapiro, 1981a, 1981b). Burgeff and Fetz (1968) investigated the interesting phenomenon of "littoral melanism," i.e., the increasingly melanistic populations of Zygaena species at some points and regions along the Mediterranean coast between Liguria and Spain. They also did a qualitative study on the light absorption and reflection behavior of more and less melanistic forms showing that melanistic forms indeed absorb more light and, hence, warm up faster. Hofmann and Tremewan (2017) reported that melanism occurs more strongly in males than in females, at least in the case of Zygaena species. Others showed that the wings are used to regulate the body temperature of butterflies (e.g., Tsai et al., 2020; Wasserthal, 1975) and that climate change has favored light‐colored insects (e.g., Zeuss et al., 2014). The physiological mechanisms of heat transfer from the wings to the body were investigated, e.g., by Liao et al. (2019).

Since the experiments of Standfuss (1900, 1901), the small tortoiseshell (*Aglais urticae*) has been underutilized in thermobiological investigations. In contrast to the wide distribution and high abundance of this species, we only found a few occasional remarks on selected webpages (e.g., https://www.flickr.com/photos/17813989@N00/7596577978/ and https://ukbutterflies.co.uk/aberrations.php?species=urticae) and in local German journals (e.g., Settele, 1981) which state that colder temperatures during rearing lead to darker coloration of the imagines.

*Aglais urticae* is a partly migratory species, whose locally reproducing populations in the lowlands are, at least in Central Europe, regularly augmented by individuals from areas of higher altitudes (Alps, low mountain ranges) (e.g., Reinhart et al., 2020). Notably, populations at low altitudes with warm (and increasingly warmer, due to climate change) winters decline significantly in recent years (Reinhart et al., 2020); hence, thisspecies is not only adapted to temperature and cold habitats but also definitely need them for its pre‐imaginal development.

According to www.lepiforum.org, *Aglais urticae* has evolved into eight different subspecies, one of these being the northern Scandinavian ssp. *A*. *urticae polaris* (Staudinger, 1871) which has a significantly darker coloration (see also, e.g., Henriksen & Kreutzer, 1982). This intensified pigmentation is most likely genetically fixed, as Aumayer (2000) was able to show in his breeding experiments that this Nordic subspecies developed significantly more dark areas on its wings even when the larvae did pupate under non‐Scandinavian, but central‐European weather conditions. Leraut (2016) mentions even more forms and subspecies and notes that the ssp. polaris is also found in Scotland, which has similar climatic conditions as Lapland. In general, the size of the markings and the intensity of coloration described by Leraut (2016) are related to the area where the forms and subspecies are described from.

This short literature compilation clearly shows that (1) butterflies can evolve genetically determined color and pigmentation pattern variations depending on the long‐term climate they live in and (2) these colors can be modified by short‐term temperature‐based climatic variations. These are important questions in the context of polyphenism which has been reviewed for insects, e.g., by Brakefield and Frankino (2009).

From theoretical considerations, it is plausible that darker individuals gain a selective advantage in cold habitats and, hence, there is a temperature‐related selection pressure in these habitats. However, it has not yet been shown whether (1) darker‐colored individuals of *A*. *urticae* have any relevant thermobiological advantage at all and (2) whether the influence of temperature during individual development determines such a quantitative shift in pigmentation intensity within the range of phenotypic plasticity that would allow a response to the thermal selection pressures at some level. In our study, we specifically addressed these two aspects of the thermobiology of butterflies.

## 2 MATERIALS AND METHODS

### 2.1 Collection and exposure of larvae and pupae

For the temperature experiments in 2020, almost fully grown larvae (L4 and L5) of *A*. *urticae* were collected from three nests adjacent to each other near Isny, Southern Germany (168 larvae in total). In 2021, we collected the same larval stage from about five nests at two locations (near Konstanz and near Stuttgart, Southern Germany, 182 larvae in total). All locations are located at a geographical altitude between 400 and 700 meters above sea level (m a.s.l.). In addition, we collected 3, 10, and 7 larvae of *A*. *urticae* at Muonio (Finland, about 250 m a.s.l., more than 300 km away from the coast of the Arctic Ocean), Alta (Norway, about 5 m a.s.l., very close to the coast of the Arctic Ocean), and Abisko (Sweden, about 380 m a.s.l., more than 50 km away from the nearest fjord coast and more than 150 km away from the coast of the open Atlantic Ocean), respectively, at the beginning of July 2020. All these Scandinavian locations are far north of the Polar Circle lying within the distribution area of the subspecies *A*. *urticae polaris*.

**FIGURE 1:**
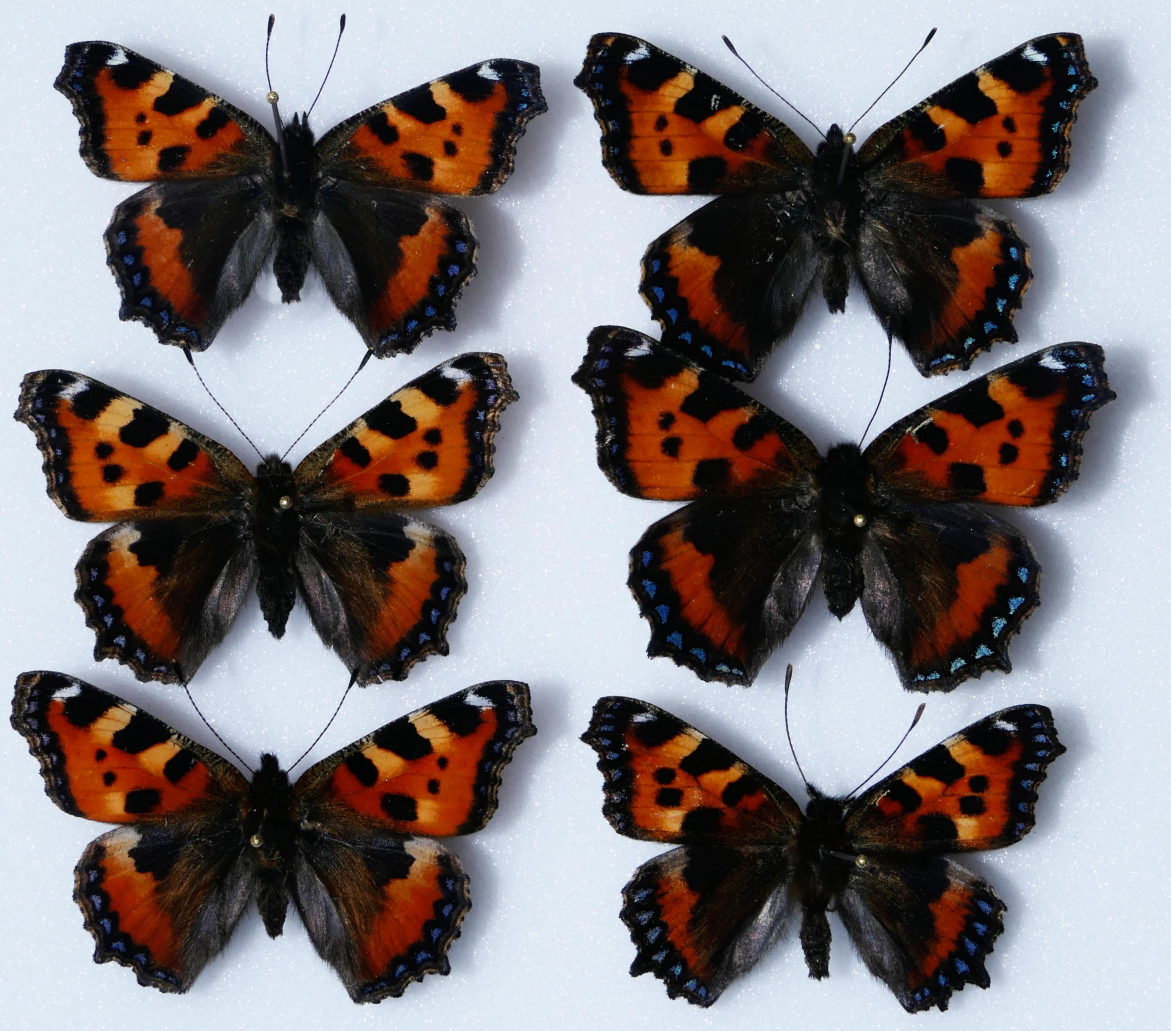
Selected mounted specimens of the Small Tortoiseshell, *Aglais urticae*, to show the variations in colors and markings among the samples whose pigmentation and heating were individually quantified

The Scandinavian larvae were reared on leaves and stalks of the stinging nettle, *Urtica dioica*, in a camping bus during the return journey to the south and they pupated all within 2 weeks following collection. They spent the majority of their pupation period in Southern Sweden and Germany in warm summer climate. They emerged between July 13 and 17, 2020 and were killed and mounted immediately after emergence (Figure 1).

In 2020, the Southern German larvae were collected on May 15, and all individuals pupated between May 22 and May 24. Until then, they were kept in a fully shaded location in Tübingen, Southern Germany, and fed with *U*. *dioica* until pupation. Starting May 26, the pupae were divided into the following six experimental groups and were, until their emergence, subjected to constant room temperature in cooling and heating cabinets at the University of Tübingen: 26 pupae at 7°C (which all failed to develop or emerge at that low temperature), 26 pupae at 11°C, 24 pupae at 20°C, 29 pupae at 29°C, and 27 pupae at 34°C (all hereinafter referred to as “lab” groups). In addition, 1 “open‐air group” of 36 pupae remained in the same location the caterpillars had pupated in and thus were subjected to typical Tübingen summer climate. Of the latter, 26 emerged on June 1, and the others on June 2. The other five groups from the climate cabinets emerged partly on the same date, partly much later, depending on the ambient temperature (Table 1). All freshly emerged butterflies were euthanized by rapid cooling and subsequent freezing, mounted to display their maximum wing size, photographed (Figure 1), and subjected to pigmentation quantification of the dorsal side and subsequent thermal experiments.

**TABLE 1:** Chronological structure of the exposure experiments

Treatment	Exposure time	Time span of emergence	Time outdoors after pupation (days)	Time in the climate cabinet after pupation (days)
2020 control group Tübingen Steinlach (15.8°C)	15 May–02 June 2020	01–02 June 2020	18	0
2020 group Alta, Norway		13–17 July 2020	21	0
2020 group Muonio, Finland		13–17 July 2020	25	0
2020 group Abisko, Sweden		13–17 July 2020	22	0
2020 group 7°C LTE	15 May–23 July 2020	13–23 July 2020	0	>50
2020 group 11°C LTE	15 May–26 June 2020	11–26 June 2020	0	36
2020 group 20°C LTE	15 May–31 May 2020	30–31 May 2020	0	9
2020 group 29°C LTE	15 May–30 May 2020	29–30 May 2020	0	8
2020 group 34°C LTE	15 May–30 May 2020	28–30 May 2020	0	8
2021 control group Tübingen Morgenstelle (15.4°C)	18 May–11 June 2021	05–11 June 2021	25	0
2021 group 11°C LTE	18 May–09 August 2021	10 July–9 August 2021	0	77
2021 group 20°C LTE	18 May–15 June 2021	03–15 June 2021	0	23
2021 group 29°C LTE	18 May–13 June 2021	04–13 June 2021	0	16
2021 group 11°C PE	18 May–23 June 2021	14–23 June 2021	25	8
2021 group 20°C PE	18 May–24 June 2021	12–24 June 2021	19	9
2021 group 29°C PE	18 May–24 June 2021	09–24 June 2021	23	8

Abbreviations: LTE, long‐term exposure; PE, pulse exposure.

**FIGURE 2:**
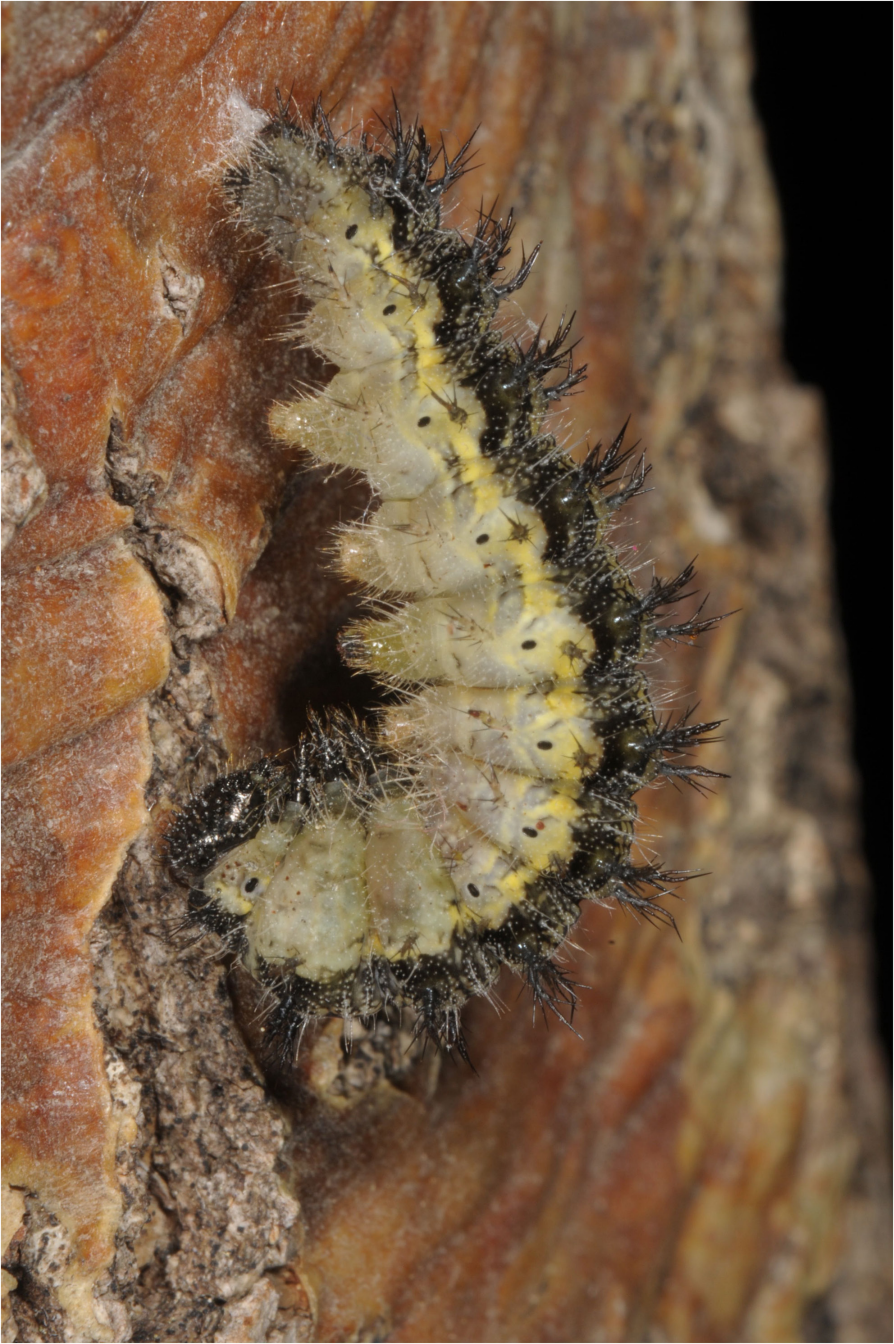
Pre‐pupa of *Aglais urticae* at the stage at which the animals were introduced into the 2021 exposure trials

In 2021, the experimental design was similar, but modified in two ways. After removal from nature, the collected larvae were distributed into 7 different groups of 26 larvae. When about 75% of the individuals in a given group showed pre‐pupa behavior (see Figure 2), this particular group was subjected to different experimental setups: either to a short‐term treatment (“pulse exposure lab experiment,” PE) under controlled temperature conditions of 11, 20, or 29°C for a given duration (Table 1) or to a long‐term lab experiment (LTE) under these temperature conditions, terminated with the emergence of the last imagines. The PE pupae were, after they were removed from the controlled temperature devices, subjected to natural conditions at the same location in Tübingen the open‐air group had been maintained in. This location was a different one from that of 2020 and occasionally exposed to full sunlight in the afternoon. The dates of emergence are displayed in Table 1. Again, all imagines were killed after emergence, mounted (Figure 1), photographed, and the dorsal pigmentation intensity quantified. No thermal experiments were carried out on the 2021 imagines.

### 2.2 Quantification of pigmentation

**FIGURE A1:**
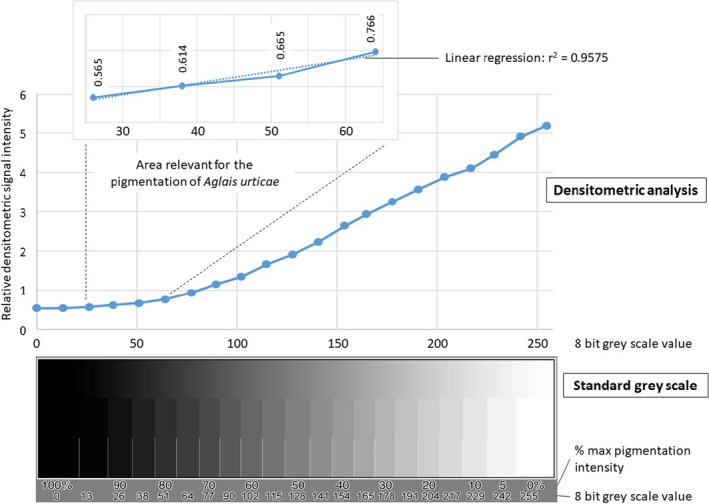
Densitometric image analysis of a standard 8 bit gray‐scale image. Standard scale (bottom) and data corresponding to the gray values, collected in the same way as for the gray value determination of the pigmentation of *Aglais urticae* (top). Linearity of data within the range of gray values relevant for *A*. *urticae* was assured by linear regression analysis with *r* ^2^ = .9575

All mounted adult butterflies were illuminated on a white plate with four natural spectrum bulbs arranged in a square and photographed with a digital camera (Sony Cyber Shot AVCHD Exmor R 18.2). According to the procedure of Davis et al. (2004) developed to quantify pigmentation in butterfly larvae, the color photos taken were then converted into grayscale using GIMP. Based on these converted images and their average 8 bit pixel brightness (PB), the average pigmentation intensity (PI) of each individual was quantified with Image J on a scale from 0 (white) to max. 255 (black) according to PI = (255 – PB). Linearity of data within the range of gray values relevant for *A*. *urticae* was assured by the analysis of images from a standardized 8 bit gray scale (see Figure A1 in the Appendix).

### 2.3 Thermobiological relevance of pigmentation

**FIGURE 3:**
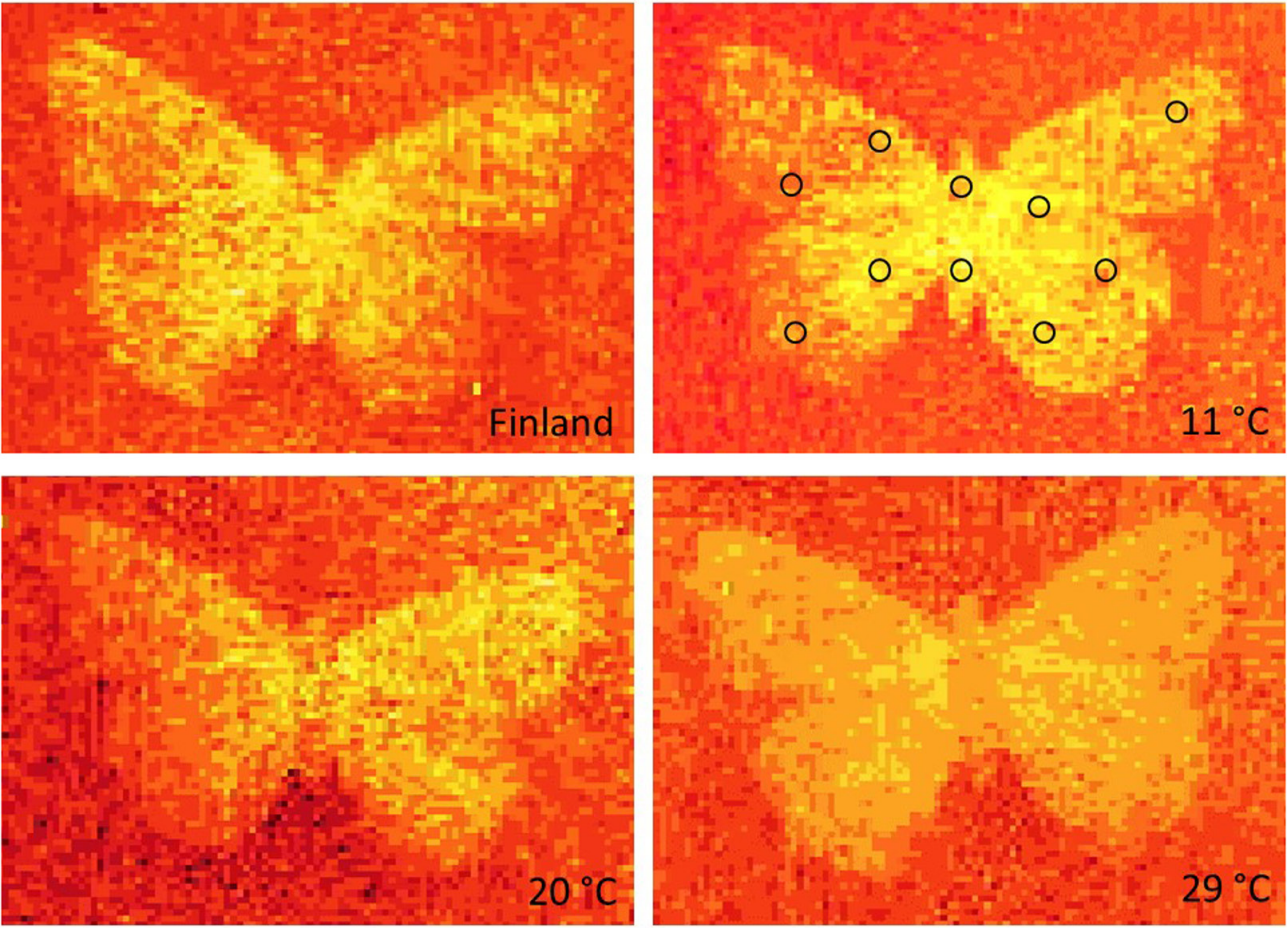
Exemplary thermographies of imagines of *Aglais urticae* after 5 min of illumination. Specimens from Scandinavia (Finland) and Central Europe (from 11°C, 20°C, and 29°C pupa exposure groups). Surface temperatures are depicted in false colors and decrease in the order bright yellow, yellow, orange, red, dark red, and brown. The average surface temperature of every individual was calculated as the mean value from 10 individual measurements at the positions on the body and wings of the animals that have been marked by circles in the upper right picture

The heating of the dorsal side of every individual with constant illumination by a light source with a natural spectrum (12 W, 1000 Lm, 5300 K) was carried out in a specifically manufactured cuboid illumination cabinet, which was closed on all sides with black wall, floor, and ceiling panels during the measurements. To equilibrate and standardize temperature at the start of the experiment, four mounted *Aglais* individuals each were fixed evenly on a white plate, and this arrangement was set to a temperature of 20°C for at least 15 min in a climate chamber. The background temperature in the lighting cabinet was 27°C at the beginning of each measurement. After placing the arrangement with the four butterflies in the lighting cabinet, they were illuminated for 5 or 15 min. After these times, thermographic images were taken with a Micro‐Epsilon thermo Imager TIM 400 thermocamera, and the surface temperature of the dorsal side was then determined using the corresponding TIM software (Figure 3) following the methodology of Köhler et al. (2021). For this purpose, 10 individual measurements were taken per animal at different but specific locations on the wings, from which an individual‐based mean value was calculated.

### 2.4 Statistics

To check for the relation between the year of investigation and the type of experiment (LTE vs. PE) and the pigmentation intensity (PI), we performed multiple regression analyses on both raw data and averages. Similarly, a multiple regression model was built to test the significance of illumination time, development temperature, and the interaction of these two parameters with the surface temperature of the dorsal side of the butterflies. Subsequently, data were subjected to linear regression analysis and 95% confidence interval calculation using JMP. Significance at the .05 level was determined by anova after checking normal distribution and homogeneity of variance. All modeling and anova were performed with JMP. A single comparison of independent mean values of pigmentation intensity of butterflies emerging from the open‐air cultures was done using Student's *t* test.

## 3 RESULTS

**FIGURE 4:**
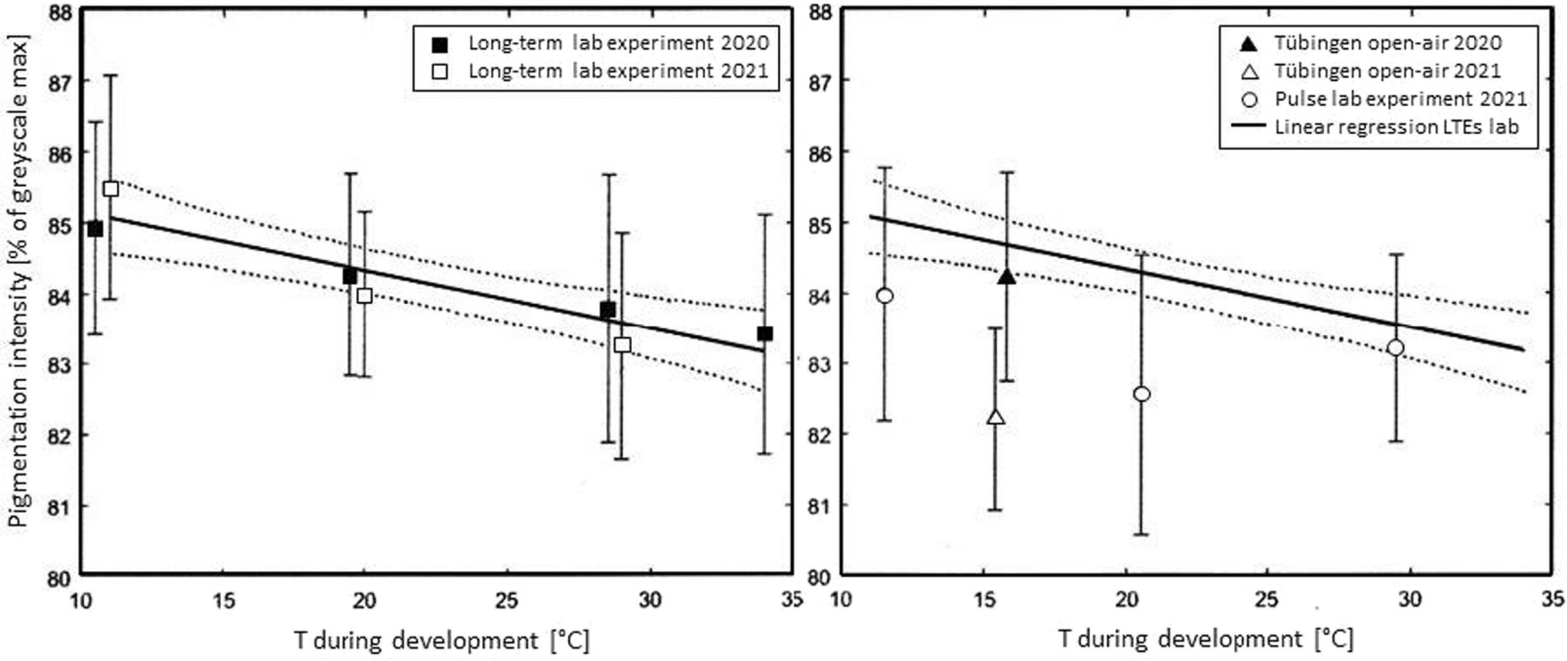
Dorsal wing and body pigmentation intensity vs. rearing temperature during pupal (and, in 2021, late larval) development. Data (means and standard deviations) from long‐term (LTE, left) and pulse lab experiments (PE, right) plus outdoor “open air” caging at Tübingen (right). Linear regression analysis (and 95% confidence intervals, dotted curves) was performed for LTE data (open and filled squares, on the left) solely and shown on the right only to visualize the differences from the other exposures. The correlation of the two parameters in the LTE was significant at *p* = .0025

Multiple regression modeling of pigmentation intensity clearly revealed the significance of the parameters “rearing temperature” and “type of experiment” (LTE vs. PE), and insignificance of the parameter “year.” Modeling the mean pigmentation intensity (*r* ^2^ = 0.78742, *n* = 10, Δx = 9, F = 7.4082, *p* = .0193), the rearing temperature (*p* = .0086), and the type of experiment (*p* = .0461) contributed significantly, but the year of investigation (*p* = .7761) did not. Following a backward deletion procedure and omitting the parameter “type of experiment,” the resulting model with borderline non‐significance (*r* ^2^ = .564791, *n* = 10, Δx = 9, F = 4.5421, *p* = .0544) still revealed significance of the rearing temperature (*p* = .0232) and non‐significance of the year (*p* = .1947). When modeling the variation in the raw data for pigmentation intensity (*r* ^2^ = .165669, *n* = 118, Δx = 117, F = 11.4175, *p* < .0001, for single year exclusively as PE was only carried out in 2021), also the contribution of both the rearing temperature (*p* = .0002) and the type of experiment (*p* = .0023) was found significant. Our experiments thus provided clear linear relationships between the rearing temperature and the average degree of darkness of the emerging imagines (Figure 4).

All data are summarized in Table 2. Significant differences in coloration existed between the various types of experiments and groups. The results of these experiments showed – to the best of our knowledge for the first time in a quantitative way – the qualitatively well‐known fact that colder temperatures during pupation lead to the emergence of darker imagines.

**TABLE 2:** Body pigmentation and thermography

Treatment	Number of individuals	Mean pigmentation (%)	Standard deviation (%)	Mean temperature after 5 min (°C)	Standard deviation (°C)	Mean temperature after 15 min (°C)	Standard deviation (°C)
2020 control group Tübingen S. (15.8°C)	30	84.22	1.48	27.5574	0.1302	28.0360	0.3536
2020 group Alta, Norway	10	84.63	1.82	27.6264	0.1492	28.2483	0.0231
2020 group Muonio, Finland	3	87.34	0.59	27.8136	0.0186	28.1742	0.0507
2020 group Abisko, Sweden	7	86.98	1.38	27.9221	0.0775	28.0818	0,1017
2020 group 11°C LTE	22	84.93	1.50	27.6260	0.2729	28.1009	0.2649
2020 group 20°C LTE	24	84.27	1.43	27.6791	0.0961	28.0660	0.1763
2020 group 29°C LTE	24	83.78	1.90	27.5700	0.2231	28.1378	0.2371
2020 group 34°C LTE	14	83.42	1.71	27.4558	0.0961	28.0802	0.0810
2021control group Tübingen M. (15.4°C)	23	82.21	1.28	n.d.	n.d.	n.d.	n.d.
2021 group 11°C LTE	15	85.50	1.57	n.d.	n.d.	n.d.	n.d.
2021 group 20°C LTE	20	83.99	1.18	n.d.	n.d.	n.d.	n.d.
2021 group 29°C LTE	24	83.25	1.60	n.d.	n.d.	n.d.	n.d.
2021 group 11°C PE	18	83.98	1.79	n.d.	n.d.	n.d.	n.d.
2021 group 20°C PE	21	82.56	1.99	n.d.	n.d.	n.d.	n.d.
2021 group 29°C PE	19	83.20	1.33	n.d.	n.d.	n.d.	n.d.

Abbreviations: LTE, long‐term exposure; M, Tübingen site “Morgenstelle”; PE, pulse exposure; S, Tübingen site “Steinlach”.

With increasing rearing temperature (T_rearing_) in the LTEs, the coloration successively became lighter in a linear relationship with the equation

$$\text{PI}=‐0.0833\;T_{\text{rearing}}+85.994\;\left(\text{ANOVA}:p=.0025\right)$$

In principle, this relationship between maintenance temperature and pigmentation intensity was almost identical regardless of whether temperature control started shortly before or shortly after pupation in the LTEs in 2020 and 2021. The PE, however, resulted in pigmentation intensities intermediate between the LTEs and the open‐air groups.

**FIGURE 5:**
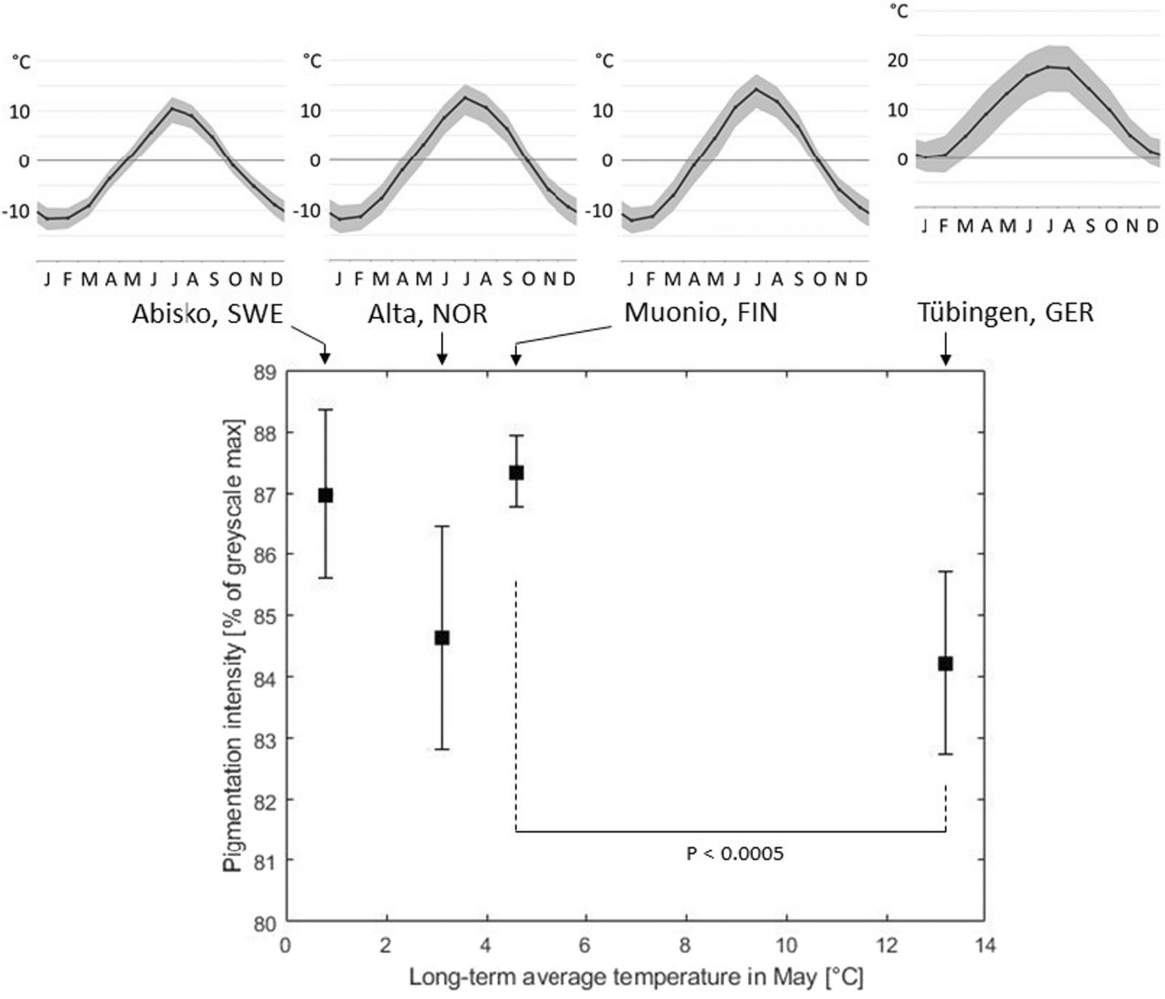
Pigmentation intensity of the imagines emerged from the open‐air treatments vs. the long‐term average temperature of the locations the respective caterpillars were taken from in May, the month of sampling. Top row: Annual temperature profiles for the four locations after https://de.climate‐data.org, modified. Single significance is given for Tübingen vs. Muonio (Student´s *t* test)

The Tübingen open‐air groups displayed noteworthy differences: while the 2020 open‐air group, which was kept in a continuously shaded place outside a building, showed a similar pigmentation intensity as the 20°C and the 29°C groups reared in climate chambers in 2020, the 2021 open‐air group was kept in a semi‐shaded place, where the full sun was allowed to reach the rearing cages in the afternoon. This group of pupae developed into imagines with the lightest coloration among all experiments, even lighter than the imagines from the lab experimental group continuously exposed to 29°C in 2021. Interestingly, the darkest imagines emerged from the Finnish and Swedish individuals without regulated temperature treatment, although they were not reared in cold climate but (mostly, starting only a few days after collection) under Central European conditions (Figure 5). The Norwegian larvae also developed into quite dark imagines showing a pigmentation intensity similar to the 11°C group of our rearing experiments in the lab.

**FIGURE 6:**
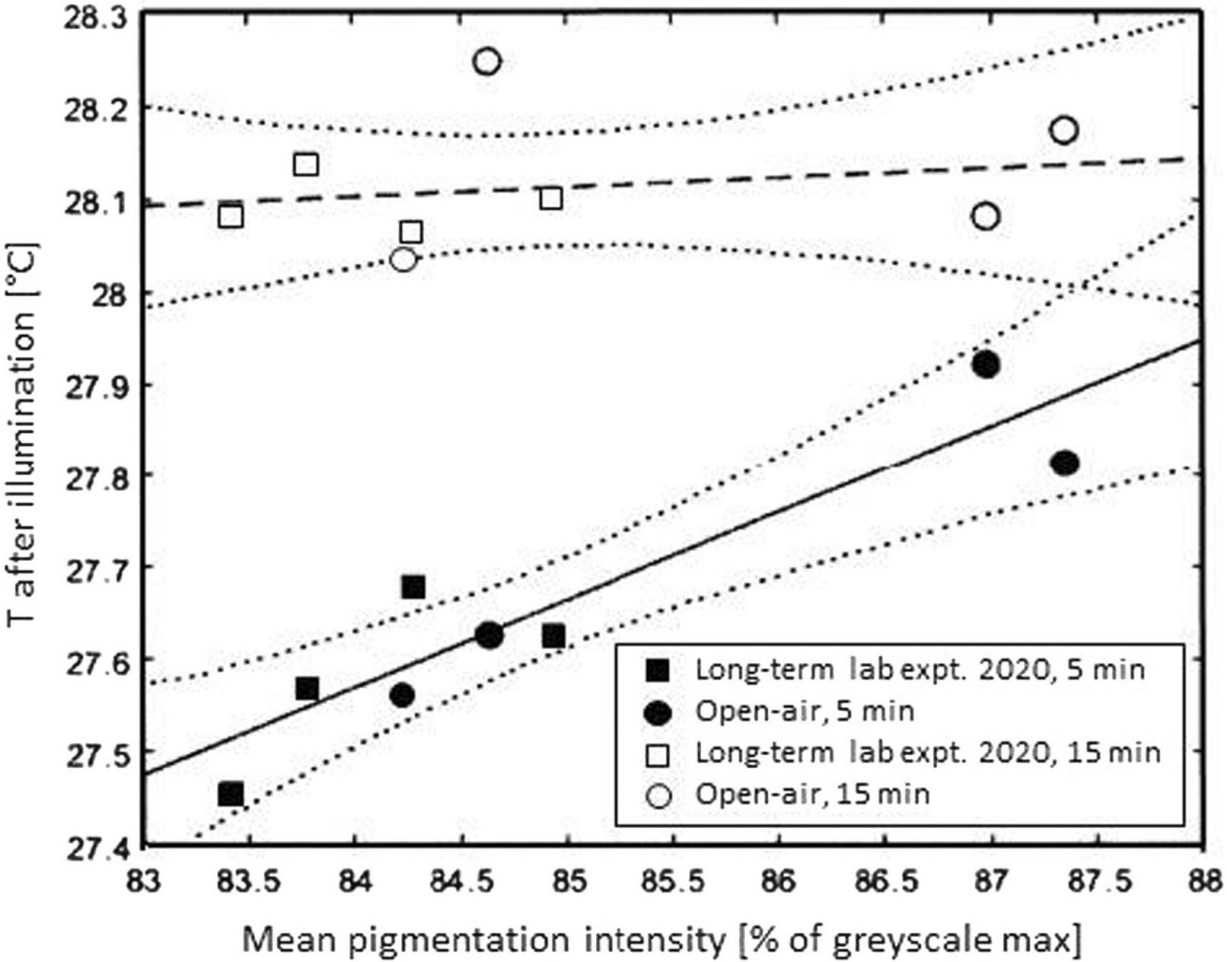
Body surface temperature vs. pigmentation intensity of the dorsal body surface in imagines emerged from LTEs (squares) and open‐air exposures (circles). Data for 5 min (black symbols) and 15 min (open symbols) of illumination. Linear regression analyses (and respective 95% confidence intervals, dotted curves) were performed for 5 and 15 min data separately. The correlation of the two parameters was significant for 5 min illumination at *p* = .0011. After 15 min illumination, body surface temperatures of differently pigmented butterflies failed to follow a significant trend (*p* = .6076)

When modeling the body temperature after illumination (*r* ^2^ = .613598, *n* = 194, Δx = 193, F = 75.0321, *p* < .0001), we found the time of illumination (*p* < .0001), the rearing temperature (*p* = .0484), and the interaction of both parameters (*p* = .0283) significant. The subsequent regression analysis revealed a linear relationship of the degree of butterfly darkness and the corresponding body temperature upon irradiation, particularly after short‐time illumination (Figure 6). Thus, the illumination experiments demonstrated that darker imagines reached higher body surface temperatures (T_body_) after 5 min of irradiation according to the linear equation

$$T_{\text{body}}=0.0949\;\text{PI}+19.598\;\left(\text{ANOVA}:p=.0011\right)$$

This effect leveled out after 15 min of irradiation at a higher temperature level when there was no longer a significant dependence of body temperature on pigmentation intensity:

$$T_{\text{body}}=0.0102\;\text{PI}+27.246\;\left(\text{ANOVA}:p=.6076\right)$$

## 4 DISCUSSION

The experimental design of the present study provides a consistent image of the relationship among environmental conditions, the morphological variation in butterfly pigmentation, and the thermobiological consequences of these color variations. Hence, this study sheds light on the ecological implications of the developmental consequences of lower habitat temperatures, which enable the resulting imagines to make better use of low light radiation in cold environments. This implies that the underlying reaction norms have evolved in response to strong thermobiological selection pressures (adaptive phenotypic plasticity). All these aspects will be discussed below.

### 4.1 Modification in coloration within the boundaries of phenotypic plasticity

The two long‐term lab experiments (LTE) from 2020 and 2021 clearly show that there is a linear relationship between the temperature the pupae are subjected to during their pupation period and the coloration of the imagines. Interestingly, and in contrast to Standfuss (1900, 1901), who proposed that coloration is crucially dependent on the external temperature during the transition from larva to pupa, the pulse experiments (PE) in the lab did not result in the same coloration and not even in a linear relationship between pulse exposure temperature and coloration (Figure 4), as the type of experiment (LTE vs. PE) proved to be a significant parameter in the multiple regression analysis. Rather the PE resulted in reduced pigmentation intensities compared with those of the LTEs, suggesting that higher temperatures in late pupal life have impeded the deposition of pigment. We conclude from these observations that, at least in *A*. *urticae*, individual coloration intensity is phenotypically plastic within a reaction norm, is ontogenetically determined during the very beginning of the pupation period (see also Otaki, 2008), but can be substantially modified during the pupal stage depending on the respective weather conditions in a given period of a specific year.

### 4.2 The importance of peak vs. average temperature during rearing

Both open‐air groups were kept outside buildings in Tübingen in 2020 and 2021, hence, they were both subjected to average Tübingen climate conditions (in which according to the German Meteorological Service, the average temperature in June is 15.3°C). However, in 2020, they were kept in the shadow continuously, while in 2021, their cages were in the shadow until about early afternoon and subsequently in full sunlight for some hours. The fact that the results of the LTEs were strikingly similar in both years suggests that the experimental animals, although deriving from different clutches from different localities in southern Germany, must have had very similar reaction norms for pigmentation intensity. Thus, the observation that the sun‐lit 2021 open‐air group resulted in the lightest imagines of all experimental groups within the 2 years (their colors reflecting the consequences of continuous temperatures above 29°C) suggests that it is not only the average temperature (which would relate to the local climate) but also the peak temperature(s) prevailing during the pupation period or illumination intensity that ultimately determine(s) the coloration intensity of the imagines.

Furthermore, it is interesting to note that even the shaded open‐air group of 2020 exhibited pigmentation intensities considerably lower (reflecting something between 20 and 29°C) than would have been expected, if they had reflected the average Tübingen June temperature of 15.3°C. This was probably a consequence of the immediate proximity of the locality to the shaded, heat‐absorbing building, which may have artificially elevated the local microclimate.

### 4.3 The interrelationship among rearing temperature, genetic predisposition, and imaginal pigmentation intensity

Our study provides three observations that are important to understand the interconnections among (i) genetic predisposition, (ii) rearing temperature during the pupal stage, and (iii) the duration of a period at a given temperature during development.

The first observation was discussed in the previous section; it is the difference in pigmentation intensity in the open‐air groups vs. the PE and LTE groups. The second observation is the fact that two of the Scandinavian groups of larvae developed into by far the darkest imagines, in spite of them not being subjected to particularly cold temperatures during the pupal period. It can be assumed that their phenotypic plasticity, defined by heritable reaction norms, is calibrated further in the direction of darker body coloration than in the sampled Central European populations. Their default coloration, therefore, is genetically fixed at a level of stronger pigmentation, suggesting the existence of a latitudinal cline in the dark pigmentation reaction norm through central and northern Europe. Hence, it may indeed be reasonable to regard them as a specific taxon, the subspecies *A*. *urticae polaris*. The fact, though, that the Norwegian imagines were considerably lighter than the Finnish and the Swedish butterflies and only slightly darker than Central European individuals cultured at 11°C in 2020 shows that, even within this subspecies, there is some variation in default pigmentation intensity. This variation cannot be attributed to solely short‐term weather conditions, as all three Scandinavian groups were kept together under the same conditions after collecting the caterpillars. The difference between the Norwegian group on the one hand and the other two Scandinavian groups on the other hand probably lies in the fact that the Norwegian group was collected near the coast of the Arctic Ocean (<100 m away from the shore), while both other groups come from far inland (see above). As large water surfaces are well known to regulate the ambient temperature in their vicinity to milder values and they buffer extremes of both high and of low temperatures, the Norwegian imagines may have been subjected to less intense selection pressure for dark coloration in a more mellow coastal climate. This is in contrast to the Finnish and Swedish imagines, which have been subjected to the harsher and more extreme inland climate far away from the coast. Hence, there may be an inland–coastal cline superimposed on top of the north–south latitudinal one for pigmentation intensity in this species.

The third observation, finally, is that the PE and LTE in 2021 caused varying coloration of the emerging imagines. All PEs resulted in imagines which showed coloration intensity indices intermediate between the open‐air groups kept outside (which they joined after the first few days of pupal stage) and the LTE groups at the same but constant temperature lasting for the entire pupal period (see Figure 4). This clearly shows that the susceptibility of this developmental stage to phenotypic plasticity in pigment deposition is initiated around the time of pupation and continued throughout pupal life. Together with the clear and linear relationship between coloration and LTE temperature, this shows that short‐term temperature (i.e., weather in nature) conditions exert an important control on modifying the pigmentation intensity within the genetically predetermined coloration range.

Hence, the color pattern and its intensity of a specific individual of *A*. *urticae* are the consequence of a complex interplay of genetic predisposition selected in response to the average temperature profile in a specific locality, and the actual mean and peak temperatures experienced in the context of oscillating weather conditions during pupal life.

### 4.4 Physiological relevance and microevolutionary implications of coloration intensity

It is a general rule that the pigmentation of the body surface plays a significant role in thermoregulation of ectotherms (Clusella Trullas et al., 2007). For many taxa, it is accepted that dark‐colored individuals absorb electromagnetic radiation more strongly and thus appear in cooler regions or cooler seasons to a greater extent, e.g., in amphibians, reptiles, snails, and insects, including butterflies (e.g., Burgeff & Fetz, 1968; Chaput‐Bardy et al., 2014; Clusella Trullas et al., 2009; Hofmann & Tremewan, 2017; Kettlewell, 1973; Laakso et al., 2021; Rudh & Qvarnström, 2013; Schweizer et al., 2019). Nevertheless, direct thermographic studies on body surfaces, especially of butterflies, are still very rare and if they exist, they are rather qualitative (e.g., Burgeff & Fetz, 1968). A commendable exception is the work of Jarvi et al. (2019), who were able to show by infrared imaging that red‐colored imagines of *Junonia coenia*, which appear in autumn, warm up faster and more strongly than tan individuals of summer. However, the actual thermobiological relevance of darker coloration of butterfly wings for ecophysiological performance remains unclear, since Rosa and Saastamoinen (2020) found that thermobiological behavior (“wing vibrating” preceding flight) in *Melitaea cinxia* was not dependent on pigmentation intensity. On the contrary, heavily pigmented individuals were more mobile in this study, which could well have had thermobiological causes. Our study showed a direct correlation between pigmentation intensity and stronger heating of wings and body in *A*. *urticae* in the first minutes of light irradiation (Figure 5). This also included individuals of the Scandinavian subspecies *A*. *u*. *polaris*. Since darker morphotypes also generally predominate in other butterfly species in cooler regions (e.g., Stelbrink et al., 2019), we assume that the darker body coloration and, associated with this, higher body temperatures even after brief irradiation, represents a selection advantage. This would especially be true in regions with lower or low‐frequency solar radiation, having led – in the long term – to the establishment of a darker subspecies. This intensified warming allows stronger pigmented animals, at least theoretically, to become active again earlier in the day or generally after periods of lower solar radiation, during which cooling of the body inevitably occurs in cold regions. Moreover, in habitats subjected to intermittent sunshine, specimens which warm up more quickly will gain a clear advantage in being able to exploit short periods of sunshine while specimens that take longer to warm up will not. Such an effect was shown by Roland (1982) and Guppy (1986) for various high mountain *Colias* and for *Parnassius phoebus* butterflies. Additional and sex‐specific advantages of more melanistic forms at high altitudes were shown for *Colias* species by Ellers and Boggs (2004). In other habitats, however, stronger melanization could cause fitness costs and reduced mating success (Hofmann & Tremewan, 2017; Kettlewell, 1973; Rosa & Saastamoinen, 2020), which plausibly could contribute to stabilizing selection of moderately pigmented individuals in temperate climates. Interestingly, Heinrich (1986) could show in a high‐mountain habitat that thermoregulation is also strongly correlated with butterfly thorax size. We believe that our study provides a more quantitative support to the hypothesis of Burgeff and Fetz (1968) who concluded for the special case of "littoral melanism" that it evolved as a response to climate change in glacial refugia in the Mediterranean region and, hence, helped to survive extreme climatic conditions over a long period of time.

## 5 CONCLUSION

The present study draws a coherent picture of temperature‐induced increase in pigmentation intensity of adult butterflies and thermographic documentation of a physiological (selective) advantage of this very same dark coloration in the same species, which provides a plausible explanation for the evolution of a darker colored subspecies in cold regions. Although partial aspects of these relationships were already known, in isolated form, for various other Lepidoptera, our work illustrates this regulative system, which was obviously evolutionarily preferred in its entirety, in one species – to our knowledge for the first time.

## AUTHOR CONTRIBUTIONS

**Gregor Markl:** Conceptualization (equal); Formal analysis (equal); Investigation (equal); Methodology (equal); Project administration (equal); Resources (equal); Writing – original draft (equal). **Shannon Ottmann:** Formal analysis (supporting); Investigation (supporting); Writing – review & editing (supporting). **Tobias Haasis:** Data curation (supporting); Formal analysis (supporting); Investigation (supporting); Visualization (supporting); Writing – review & editing (supporting). **Daniela Budach:** Investigation (supporting); Methodology (supporting); Resources (supporting); Writing – review & editing (supporting). **Stefanie Krais:** Data curation (supporting); Investigation (supporting); Methodology (supporting); Writing – review & editing (supporting). **Heinz‐R. Köhler:** Conceptualization (equal); Formal analysis (equal); Investigation (equal); Methodology (equal); Project administration (equal); Resources (equal); Writing – original draft (equal).

## CONFLICT OF INTEREST

None declared.

## Supporting information

DataS1

## Data Availability

All original data of this study can be found in a Supporting Information file published with this manuscript.
